# Clinicopathological and computed tomography features of patients with early-stage non-small-cell lung cancer harboring ALK rearrangement

**DOI:** 10.1186/s40644-023-00537-y

**Published:** 2023-02-23

**Authors:** Xiaoming Hou, Han Chen, You Liu, Sandong Gong, Meizi Zhudai, Leilei Shen

**Affiliations:** 1 Department of Radiology, Hainan Hospital of PLA General Hospital, Sanya, 572013 China; 2 Department of Information, Hainan Hospital of PLA General Hospital, Sanya, 572013 China; 3 Department of Pathology, Hainan Hospital of PLA General Hospital, Sanya, 572013 China; 4 Department of Gastroenterology, Hainan Hospital of PLA General Hospital, Sanya, 572013 China; 5 Department of Thoracic Surgery, Hainan Hospital of PLA General Hospital, Jiang-Lin Road, Hai Tang District, Sanya, 572013 China

**Keywords:** Anaplastic lymphoma kinase, NSCLC, Computed tomography, Genomic mutation status

## Abstract

**Background:**

Although some studies have assessed the correlation between computed tomography (CT) features and anaplastic lymphoma kinase (ALK) rearrangement in patients with non-small-cell lung cancer (NSCLC), few have focused on early-stage patients. The results of some previous studies are inconsistent and contradictory. Therefore, this study aimed to analyze the clinicopathological and CT features of patients with early-stage NSCLC harboring ALK rearrangement.

**Methods:**

This retrospective analysis included 65 patients with ALK rearrangement and 629 ALK-negative patients. All patients had surgically resected NSCLC and were diagnosed with stage IA or stage IIB NSCLC. Clinicopathological features and CT signs, including tumor size and density, consolidation tumor ratio (CTR), lesion location, round or irregular shape, lobulated or spiculated margins, air bronchograms, bubble-like lucency or cavities, and pleural retraction, were investigated according to different genotypes.

**Results:**

The prevalence of ALK rearrangement in patients with early-stage NSCLC was 9.3% (65/694). Patients with ALK rearrangement were significantly younger than those without ALK rearrangement (*P* = 0.033). The frequency of moderate cell differentiation was significantly lower in tumors with ALK rearrangement than in those without ALK rearrangement (46.2% vs. 59.8%, *P* = 0.034). The frequency of the mucinous subtype was significantly higher in the ALK-positive group than in the ALK-negative group (13.8% vs. 5.4%, *P* = 0.007). No significant differences were found in any CT signs between the ALK-positive and ALK-negative groups.

**Conclusions:**

Patients with ALK-positive lung cancer may have specific clinicopathological features, including younger age, lower frequency of moderate cell differentiation, and higher frequency of the mucinous type. CT features may not correlate with ALK rearrangement in early-stage lung cancer. Immunohistochemistry or next-generation sequencing is needed to further clarify the genomic mutation status.

## Background

Lung cancer is the leading cause of cancer-related mortality worldwide, with non-small cell lung cancer (NSCLC) accounting for approximately 85% of all cases [1]. Most patients are staged as having locally advanced or metastatic NSCLC at the first diagnosis. Platinum-based chemotherapy, with or without radiotherapy, is the standard treatment for these patients, although the survival benefits are limited [2]. With extensive research on molecular biology, targeted therapy, particularly epidermal growth factor receptor-tyrosine kinase inhibitors (EGFR-TKIs) and anaplastic lymphoma kinase-TKIs showed greater response rates and longer OS than chemotherapy in advanced NSCLC in the last decade, and has been recommended as the first-line treatment by the National Comprehensive Cancer Network (NCCN) guideline [3–9]. Meanwhile, immune checkpoint inhibitors have transformed the algorithm for patients with unresectable or advanced NSCLC without driver genetic mutations [10]. First described in 2007 by Soda et al. [11] and Rikova et al. [12], the echinoderm microtubule-associated protein-like 4-anaplastic lymphoma kinase (EML4-ALK) rearrangement was found to be carcinogenic. In 2009, Shaw et al. [13] classified ALK gene fusion as a separate subtype of lung cancer, and now, we call it a “diamond mutation” for its estimated prevalence of 3–7% in NSCLC. More importantly, ALK-TKIs, such as crizotinib [14], ceritinib [15], alectinib [16], brigatinib [17], and lorlatinib [18], were superior to cytotoxic chemotherapy, with a higher response rate and excellent progression-free survival of approximately 8 years.

Some studies have assessed the imaging characteristics of NSCLC with ALK rearrangement or their differences from those of other gene alterations [19–24]. However, unified conclusions were not drawn for the following reasons: a small number of patients, most of the enrolled patients with advanced NSCLC had insufficient specimens obtained from biopsy samples of primary or metastatic sites, which may not accurately reflect the pathological and molecular characteristics, and limited numbers of evaluated computed tomography (CT) findings [20–23]. To the best of our knowledge, no study has been conducted to identify the clinicopathological and radiological features associated with ALK rearrangement in early-stage NSCLC.

Therefore, this study aimed to analyze ALK-positive and ALK-negative patients with early-stage NSCLC and retrospectively review the clinicopathological features and CT signs of each patient to determine the useful characteristics of different genomic statuses.

## Methods

### Study population

Between January 2017 and June 2021, 694 patients with stage IA to stage IIB NSCLC were enrolled. All the patients underwent surgical resection for primary lung cancer at the Chinese People’s Liberation Army General Hospital. Patient medical records were reviewed to extract data on clinicopathological characteristics, and all the tissues used for genomic testing were obtained from the hospital tissue bank. The patient inclusion criteria were as follows: (a) NSCLC with ALK rearrangement detected in the surgical specimens, (b) stage IA to stage IIB NSCLC, and (c) preoperative thin-section CT scan performed < 1 month before surgery. Patients who received neoadjuvant chemotherapy, immunotherapy, targeted therapy, or radiotherapy or were diagnosed with other lung cancers were excluded. Data on age, sex, smoking status, family history of lung cancer, preoperative serum carcinoembryonic antigen (CEA) level, histological subtype, and pathological TNM stage were collected for each patient. The requirement for informed consent was waived by the ethics committee of our hospital, as this was a retrospective study.

### Tumor pathology and mutation analysis

**Fig. 1:**
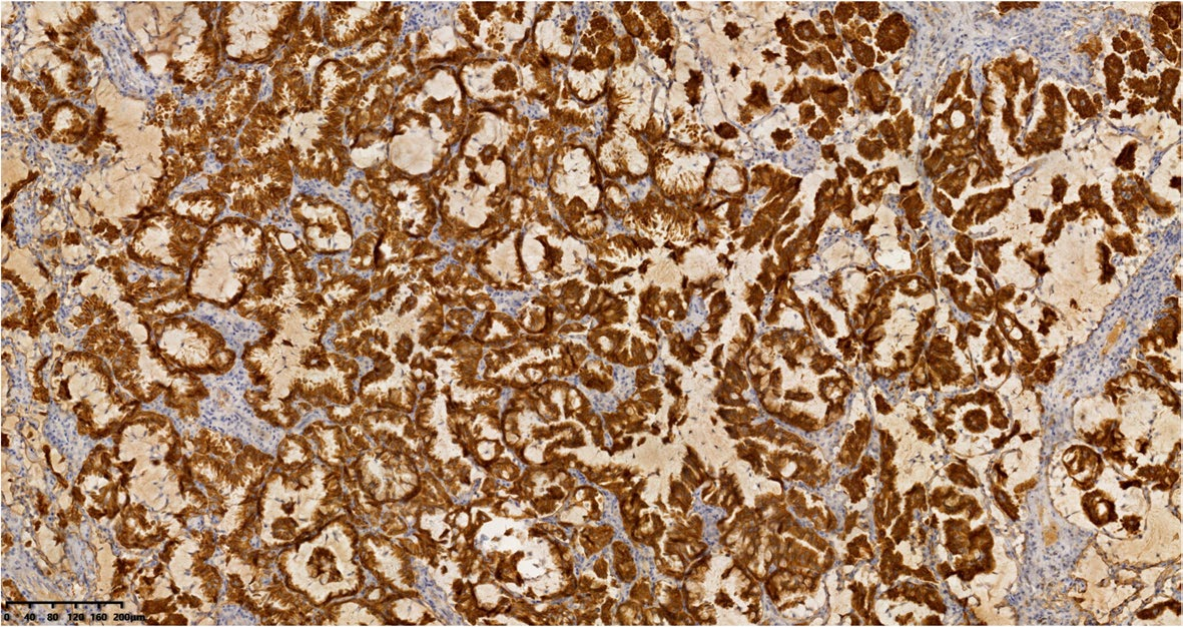
Immunohistochemistry (IHC). The VENTANA IHC assay reveals cytoplasmic ALK staining

**Fig. 2:**
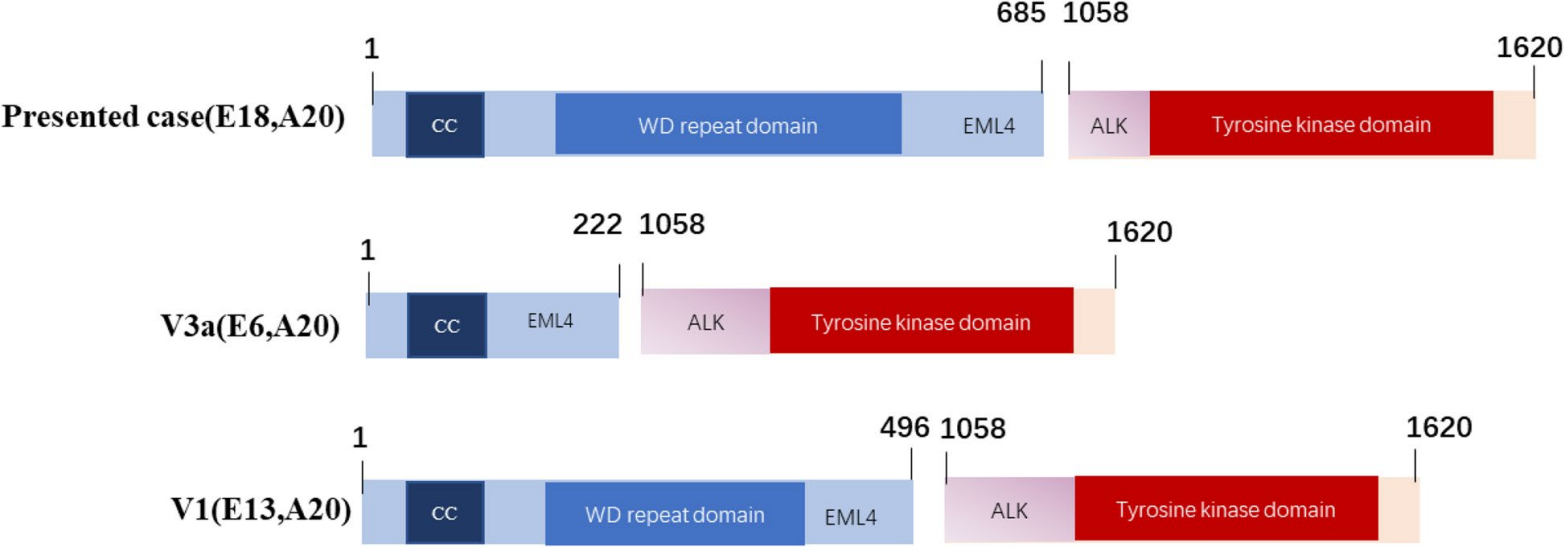
Next-generation sequencing (NGS). NGS reveals EML4-ALK fusion in the presented case compared with the two most common variants, V1 and V3

All histological and mutation analyses were performed on surgical specimens. Tumor histology was classified using the World Health Organization (WHO) criteria (2015 version) [25]. NSCLC staging was performed according to the 8th TNM staging system [26]. ALK positivity was analyzed by immunohistochemistry (IHC) (Fig. 1) or next-generation sequencing (NGS) (Fig. 2). Mutations in epidermal growth factor receptor (EGFR) and Kirsten rat sarcoma viral oncogene (KRAS) were identified using NGS. IHC for ALK protein expression was performed on formalin-fixed paraffin-embedded sections using a VENTANA ALK (Clone D5F3) CDx kit and benchmark Ultra Immunostainer (Ventana Medical Systems, Inc., Tucson, AZ, Cell Signaling Technology) according to the manufacturer’s protocols. Genecast Technology (Wuxi, China) performed the entire NGS process, and all procedures were conducted using the manufacturer’s scheme.

### CT imaging and characteristics

Chest CT examinations were performed on a Discovery CT750 HD (GE Healthcare, Milwaukee, WI, USA) scanner with the following scanning parameters: 120 kVp, 150–200 mA, and 1.25 mm reconstruction thickness with a 1.25 mm reconstruction interval. Two radiologists (X. M. Hou and H. Chen, with 10 and 5 years of experience in chest CT diagnosis, respectively) independently reviewed the images. They were aware that all patients had been diagnosed with NSCLC but were blinded to the genomic phenotype status. The following CT characteristics were assessed: tumor size, tumor density (solid/ground-glass opacity), consolidation tumor ratio (CTR = consolidation size/tumor size), lesion location (central or peripheral), round or irregular (spherically shaped lesions with smooth borders or not), lobulated or spiculated margins, air bronchograms, bubble-like lucency or cavities, and pleural retraction. The ground-glass opacity (GGO) included pure GGO and mixed GGO and was assessed by the CTR.

### Statistical analysis

All statistical analyses were performed using SPSS version 22 software (IBM Corporation, Armonk, NY, USA). Comparisons between ALK-positive and ALK-negative patients for categorical variables were performed using the chi-squared test or Fisher’s exact test. Differences in continuous variables (age and quantitative CT features) between ALK-positive and ALK-negative patients were tested using the Mann–Whitney U test. A two-sided *p*-value < 0.05 was considered statistically significant.

## Results

### Patient characteristics

The clinical characteristics of patients with lung cancer are summarized in Table 1. The prevalence of ALK positivity among patients with stage IA-IIB NSCLC was 9.3% (65 of 694 patients). The mean ages of the ALK-positive and ALK-negative groups were 55.86 ± 9.41 years (range, 33–73) and 58.55 ± 9.72 years (range, 23–81), respectively. Patients with ALK rearrangements were significantly younger than those who were ALK-negative (*P* = 0.033). There were no differences between the two groups regarding sex, smoking history, family history of lung cancer, or CEA levels.

**Table 1:** Characteristics of patients in the ALK-positive and ALK-negative groups

Variables	ALK+ (*n* = 65)	ALK- (*n* = 629)	*Χ*^*2*^*(t)*	*P*-value
Age (years) Mean ± SD (range)	55.86 ± 9.41 (33–73)	58.55 ± 9.72 (23–81)	−2.133	0.033^a^
≥ 60 y	24 (36.9)	313 (49.8)	3.887	0.049
< 60 y	41 (63.1)	316 (50.2)	3.887	0.049
Sex			0.024	0.876
Male	29 (44.6)	287 (45.6)		
Female	36 (55.4)	342 (54.4)		
Smoking			1.913	0.167
Nonsmoker	13 (20.0)	176 (28.0)		
Smoker	52 (80.0)	452 (72.0)		
Family history of lung cancer	8 (12.3)	87 (13.9)	0.119	0.730
CEA level			4.009	0.050
Normal (0–5 ng/mL)	1 (1.5)	54 (8.6)		
Abnormal (> 5 ng/mL)	64 (98.5)	575 (91.4)		

Unless otherwise indicated, data are shown as numbers with percentages in parentheses. ^a^indicates significant difference

*Abbreviations*: *CEA* Carcinoembryonic antigen

### Genomic mutations and pathological findings

Comparisons of pathological findings with different genomic mutations are shown in Table 2. Among the ALK-positive patients, mutations in EGFR were present in 13.8% (9/65), and mutations in KRAS were present in 1.5% (1/65). There was a significant difference between the two groups regarding EGFR mutations (*P* < 0.001). The frequency of moderate cell differentiation was significantly lower in tumors with ALK rearrangement than in those without it (46.2% vs. 59.8%, *P* = 0.034). The frequency of the mucinous subtype was significantly higher in the ALK-positive group than in the ALK-negative group (13.8% vs. 5.4%, *P* = 0.007). There were no differences between ALK-positive and ALK-negative patients regarding pathological type, micropapillary/solid subtype, VPI, lymphovascular invasion, STAS, Ki-67, PD-1, PD-L1, KRAS mutation, or TP53 mutation. Of the 65 ALK-positive patients, 34 (52.3%) had stage IA disease, 30 (46.2%) had stage IB disease, and 1 (1.5%) had stage IIB disease. No significant differences were observed in the pathological stage between the two groups.

**Table 2:** Pathological outcomes of the ALK-positive and ALK-negative groups

Variables	ALK+ (*n* = 65)	ALK- (*n* = 629)	*Χ*^*2*^*(t)*	*P*-value
Pathological type			3.240	0.101
SCC	0 (0)	30 (4.8)		
ADC	65 (100)	599 (95.2)		
Cell differentiation				
Well	16 (24.6)	123 (19.5)	0.942	0.332
Moderate	30 (46.2)	376 (59.8)	4.504	0.034^a^
Poor	19 (29.2)	130 (20.7)	2.562	0.109
Mucinous	9 (13.8)	34 (5.4)	7.222	0.007^a^
≥5% Micropapillary/solid subtype	16 (24.6)	124 (19.7)	0.879	0.348
VPI	24 (36.9)	300 (47.7)	2.746	0.097
Lymphovascular invasion	0 (0)	11 (1.7%)	1.155	0.282
STAS	3 (4.6)	10 (1.6)	2.934	0.114
Ki-67 (IQR)	5 (5, 10)	7 (3, 15)	0.277	0.782
PD-1 (IQR)	8 (1, 10)	5 (2, 10)	0.844	0.399
PD-L1 (IQR)	0 (0, 2)	0 (0, 2)	0.020	0.984
EGFR	9/65 (13.8)	242/347 (69.7)	71.842	< 0.001^a^
KRAS	1/65 (1.5)	29/336 (8.6)	3.000	0.083
TP53	2/20 (10.0)	51/239 (21.3)	1.458	0.385
pTNM stage			–	1
IA/IB	34 (52.3)/30 (46.2)	275 (43.7)/343 (54.5)		
IIA/IIB	0 (0)/1 (1.5)	11 (1.8)/0 (0)		

Unless otherwise indicated, data are shown as numbers with percentages in parentheses. ^a^indicates significant difference

*Abbreviations*: *SCC* Squamous cell carcinoma, *ADC* Adenocarcinoma, *VPI* Visceral pleural invasion, *STAS* Spread through air space, *IQR* Interquartile range, *PD-1* Programmed death-1, *PD-L1* Programmed death ligand-1, *EGFR* Epidermal growth factor receptor, *KRAS* Kirsten rat sarcoma, *TP53* Tumor protein 53

### Genomic mutations and visual CT signs

**Fig. 3:**
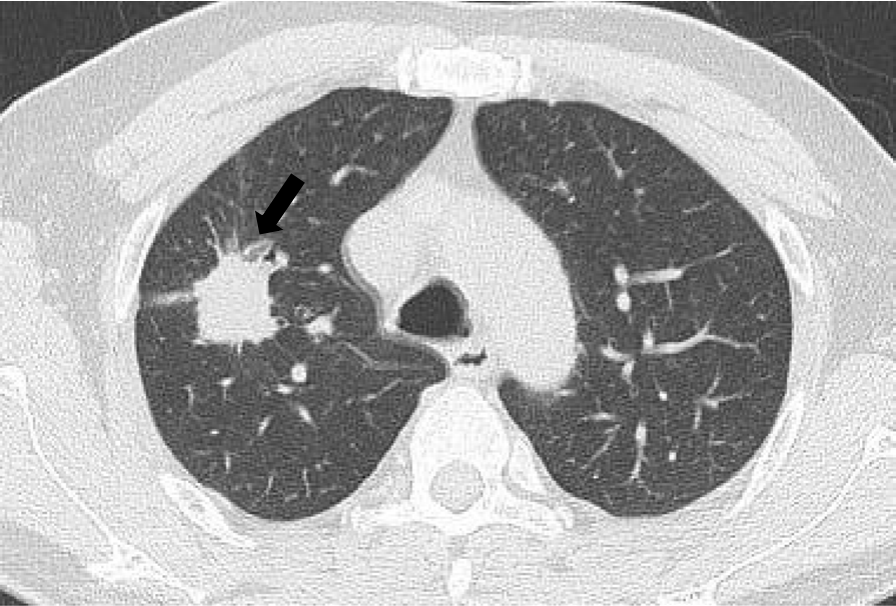
Solid nodule with ALK rearrangement. Axial CT image in a patient with ALK-positive adenocarcinoma showed a typical solid right upper lobe mass (arrow) with lobulation and spiculation

**Fig. 4:**
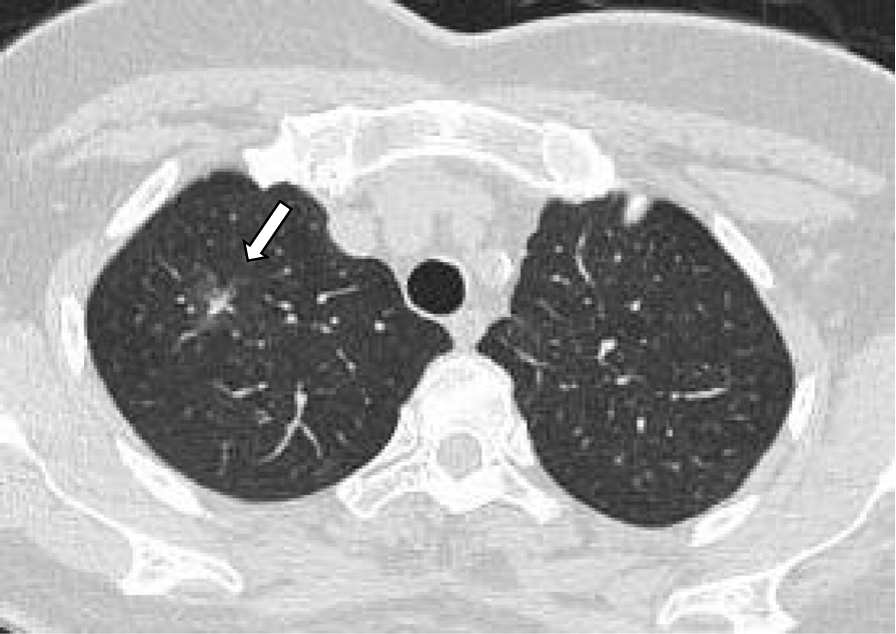
Ground-glass opacity (GGO) with ALK rearrangement. Axial CT image in a patient with ALK-positive adenocarcinoma showed a pure-GGO (arrow) in the right upper lobe with vessel underlying

The results of the visual CT signs based on different genomic alterations are summarized in Table 3. No relevant differences were observed in tumor size, location, or anatomical type between the ALK-positive and ALK-negative groups. The proportions of solid nodules (Fig. 3) and GGO (Fig. 4) were similar between the two groups. The CTR was 0.44 in the ALK-positive group and 0.66 in the ALK-negative group. No significant differences were observed in the CTR. Regarding structural characteristic features, there was no difference in the frequency of irregular borders, lobulated margins, spiculation, bubble-like lucency or cavities, pleural retraction, or air bronchograms.

**Table 3:** CT signs in the ALK-positive and ALK-positive groups

Variables	ALK+ (*n* = 65)	ALK- (*n* = 629)	*Χ*^*2*^*(t)*	*P*-value
Tumor size, mm (IQR)	15 (10, 21)	15 (12, 23)	1.101	0.271
Tumor location			–	1
Left upper lobe	17 (26.2)	147 (23.4)		
Left lower lobe	10 (15.4)	103 (16.3)		
Right upper lobe	13 (20.0)	118 (18.8)		
Right middle lobe	7 (10.8)	63 (10.0)		
Right lower lobe	18 (27.7)	198 (31.5)		
Anatomical type			1.155	0.612
Central	0 (1.7)	11 (1.7)		
Peripheral	65 (98.3)	618 (98.3)		
CT density				
Solid nodule	26 (40.0)	246 (39.1)	0.020	0.889
GGO	39 (60.0)	383 (60.9)	0.020	0.889
CTR	0.44 (0, 1.00)	0.66 (0.30, 1.00)	0.880	0.379
Irregular	43/61 (70.5)	407/547 (74.4)	0.437	0.509
Lobulated	47/61 (77.0)	402/547 (75.5)	0.360	0.549
Spiculation	44/61 (72.1)	412/547 (75.3)	0.298	0.585
Bubble-like lucency or cavities	35/61 (57.4)	270/547 (49.4)	1.411	0.235
Pleural retraction	40/61 (65.6)	418/547 (76.4)	3.472	0.062
Air bronchogram	24/61 (39.3)	164/547 (30.0)	2.252	0.133

Unless otherwise indicated, data are shown as numbers with percentages in parentheses

*Abbreviations*: *IQR* Interquartile range, *CT* Computed tomography, *GGO* Ground-glass opacity, *CTR* Consolidation tumor ratio

## Discussion

In this study, we investigated the clinicopathological and radiological features of patients with early-stage NSCLC harboring ALK rearrangement. Our findings suggest that younger patients and those with mucinous histology are prone to ALK positivity. CT features do not appear to be predictors of ALK rearrangement.

Patients with ALK-positive lung cancer have been reported to have some clinical and radiological features, such as younger age, no or light smoking history, lower GGO proportion, lobulated margins, and lymph node involvement [21]. In our study, age was the only preoperative clinical feature notably associated with ALK-positive early-stage lung cancer. Nonetheless, distinguishing ALK mutation status using age alone without IHC or genetic testing is difficult. Some studies [27–30] have shown that ALK-rearranged lung adenocarcinomas are significantly associated with the solid-predominant subtype. However, our study found no difference between ALK-positive and ALK-negative patients in the micropapillary or solid subtypes (> 5%), which is considered to be associated with poor prognosis. The difference between our results and those of others studies may be due to the high proportion (60%) of GGOs in our study, which had a lower heterogeneity than pure solid nodules. Lung adenocarcinomas are composed of complex mixtures of histologic patterns present in 5% increments, according to 2015 WHO classification of lung tumors [25]. Hence, due to intratumoral heterogeneity, the histological subtype diagnosis of lung adenocarcinoma may not be sufficient for the precise prediction of ALK rearrangement. Our study also found that mucinous-type early-stage adenocarcinoma was more common in ALK-positive patients (13.8% vs. 5.4%, *P* = 0.014), which is consistent with the report by Yoshida et al [28] They demonstrated that the mucinous cribriform pattern was present in 56% of ALK-positive tumors and in only 1% of ALK-negative tumors (*P* < 0.0001), which was confirmed to be the strongest histologic indicator of ALK rearrangement by multivariate analysis. However, the difference in mucinous types between ALK-positive and ALK-negative patients in our study was not as large as that in a previous study. Yoshida et al. [28] explained that characteristic histologies, including the mucinous cribriform pattern, are present only focally in many ALK-rearranged patients. Therefore, we believe that clinical and pathological findings are useful only to raise suspicion, particularly for early-stage NSCLC.

ALK rearrangement and EGFR or KRAS mutations have been previously reported to be mutually exclusive [13, 29, 30]. The EURTAC trial [31], published in 2012, was the first to confirm the presence of concomitant EGFR mutations and ALK rearrangement in advanced NSCLC, with an incidence of 15.8%. Similar studies [32, 33] reported that their data varied from 5 to 15% depending on the method used, and their features included female sex, East Asian population, no smoking, and stage IV adenocarcinoma. The incidence of concomitant ALK rearrangement and EGFR mutation in our study was 13.8%, and that of concomitant ALK rearrangement and KRAS mutation was 1.5%, based on NGS testing. The hypothesis of monoclonal or polyclonal origin may contribute to concomitant mutations. Cai et al. [34] found that ALK rearrangement and EGFR mutations were not from the same tumor cells and that the concomitance was due to intratumoral heterogeneity. Sasaki et al. [35] demonstrated the coexistence of EGFR signaling in an ALK-positive cell line. Further studies are needed to clarify the co-activation of these two gene alterations.

Radiogenomics is an active field for extending image features into the era of molecular imaging and has potential value in guiding appropriate genomic testing [22, 36]. Previous studies have analyzed the correlation between CT signs and ALK-positive NSCLC. Yamamoto et al. [37] found that ALK rearrangement is associated with central tumor location, absence of pleural retraction, and massive pleural effusion. In another study, Choi et al. [21] showed that lobulated margins, N2 or N3 lymph node metastasis, and lymphangitic lung metastasis were more common in ALK-positive patients. Kim et al. [38] published a meta-analysis that included 16 studies comprising 3113 patients and concluded that ALK-rearranged NSCLC was more frequently solid, central in location, and 3 cm or smaller. Another meta-analysis by Mendoza et al. [39], comprising 2210 patients, revealed that ALK rearrangements were more likely to occur in solid tumors, have a lower frequency of cavities, and a higher frequency of air bronchogram, whereas there was no significant correlation with tumor size, lobulated margins, or spiculation. Nonetheless, to the best of our knowledge, these CT signs may be found in most patients with advanced lung cancer and have no predictive value in correlating with ALK-rearranged lung cancer in the pre-advanced stages. Zhou et al. [23] demonstrated that ALK-rearranged tumors were more likely to be solid tumors without GGO components and have a lower tumor shadow disappearance rate than wild-type tumors, suggesting that a solid radiological feature is a key feature of ALK-rearranged tumors. This perspective is consistent with that of other studies on surgically resected NSCLC [19, 20, 28]. This result was also consistent with the pathological finding that the solid pattern was more common in ALK-positive tumors and pure solid nodules. The GGO component was more likely to present a lepidic growth pattern with EGFR mutation. However, our study failed to conclude this result and suggested that there was no notable correlation between ALK rearrangement of solid tumors, GGO component (or CTR), or structural characteristic features, including the frequency of irregular borders, lobulated margins, spiculation, bubble-like lucency or cavities, pleural retraction, or air bronchograms. Therefore, we believe that patients with early-stage NSCLC harboring ALK rearrangement may have no remarkable CT features.

Although there have been many studies on the correlation between CT characteristics and ALK rearrangement [19–24], it is important to note that CT signs rarely play a referential role in locally advanced or metastatic NSCLC. Our study confirms this perspective, and fewer studies should focus on this in the future. Some studies suggested that specific CT features may help to guide biopsy for further gene mutational tests [22], which is of lesser significance because of the recommendation made by the NCCN guidelines that testing for common gene mutations and immune biomarkers should be conducted in all appropriate patients [9]. The emerging radiogenomics used in NSCLC may have predictive value in the prognosis and response to treatment, including targeted therapy and immunotherapy. Regarding early-stage NSCLC, intratumoral heterogeneity changes instantly during the progression from GGO to a pure solid nodule, in which the mutational gene status and CT characteristics may also vary. Future studies should focus on this topic. In addition, a novel radiotracer may be invented to be combined with ALK, EGFR, ROS1, or other uncommon driver genes with a suspected target, such as 18F-fluorodeoxyglucose in positron emission tomography-computed tomography or TC-methylene diphosphate in the bone scan.

Our study had several limitations. First, this retrospective study conducted in a single thoracic center had relatively few enrolled patients with ALK rearrangements (65 patients). We did not perform propensity score matching to minimize the effects of observed confounders. As described previously, multicenter prospective studies with a larger number of cases are needed to verify the conclusions of early-stage NSCLC. Second, we focused on the clinicopathological and radiologic features of ALK-positive patients with surgically resected lung cancer in the early stage; therefore, our findings may not be generalizable to patients with ALK rearrangement in the locally advanced or metastatic stage. Third, we did not have follow-up information to evaluate disease recurrence or survival because of the good prognosis of patients with early-stage NSCLC. Fourth, our study analyzed ALK positivity using IHC or NGS, and this discrepancy should also be considered a confounder. Finally, interobserver variability was not thoroughly considered when CT images were evaluated.

## Conclusions

Patients with ALK-positive lung cancer may have clinicopathological features, including younger age, lower frequency of moderate cell differentiation, and higher frequency of the mucinous type. CT features may not correlate with ALK rearrangement in early-stage lung cancer. IHC or NGS is needed to further clarify the genomic mutation status.

## Data Availability

The datasets used and/or analyzed during the current study are available from the corresponding author on reasonable request.

## Abbreviations

NSCLC: Non-small cell lung cancer
ALK: Anaplastic lymphoma kinase
CEA: Carcinoembryonic antigen
CT: Computed tomography
GGO: Ground-glass opacity
CTR: Consolidation tumor ratio
IHC: Immunohistochemistry
NGS: Next-generation sequencing
SCC: Squamous cell carcinoma
VPI: Visceral pleural invasion
PD-1: Programmed death-1
PD-L1: Programmed death ligand-1
EML4-ALK: Echinoderm microtubule-associated protein-like 4-anaplastic lymphoma kinase
EGFR: Epidermal growth factor receptor
KRAS: Kirsten rat sarcoma viral oncogene
TKI: Tyrosine kinase inhibitor
TP53: Tumor protein 53
